# The Cerebellum of Patients with Steatohepatitis Shows Lymphocyte Infiltration, Microglial Activation and Loss of Purkinje and Granular Neurons

**DOI:** 10.1038/s41598-018-21399-6

**Published:** 2018-02-14

**Authors:** Tiziano Balzano, Jerónimo Forteza, Pilar Molina, Juan Giner, Ana Monzó, Jennifer Sancho-Jiménez, Amparo Urios, Carmina Montoliu, Vicente Felipo

**Affiliations:** 1 Laboratory of Neurobiology, Centro Investigación Príncipe Felipe, Valencia, Spain; 2 Instituto Valenciano de Patología, Unidad Mixta de Patología Molecular. CIPF/Universidad Católica, Valencia, Spain; 3 Instituto de Medicina Legal y Ciencias Forenses, Valencia, Spain; 4 Departamento de Patología, Facultad de Medicina, Universidad Valencia, Valencia, Spain; 5 Instituto de Investigación Sanitaria-INCLIVA, Valencia, Spain

## Abstract

Peripheral inflammation contributes to minimal hepatic encephalopathy in chronic liver diseases, which could be mediated by neuroinflammation. Neuroinflammation in cerebellum of patients with chronic liver diseases has not been studied in detail. Our aim was to analyze in cerebellum of patients with different grades of liver disease, from mild steatohepatitis to cirrhosis and hepatic encephalopathy: (a) neuronal density in Purkinje and granular layers; (b) microglial activation; (c) astrocyte activation; (d) peripheral lymphocytes infiltration; (e) subtypes of lymphocytes infiltrated. Steatohepatitis was classified as SH1, SH2 and SH3. Patients with SH1 show Th17 and Tfh lymphocytes infiltration in the meninges, microglia activation in the molecular layer and loss of 16 ± 4% of Purkinje and 19 ± 2% of granular neurons. White matter remains unaffected. With the progression of liver disease to worse stages (SH2, SH3, cirrhosis) activation of microglia and astrocytes extends to white matter, Bergman glia is damaged in the molecular layer and there is a further loss of Purkinje neurons. The results reported show that neuroinflammation in cerebellum occurs at early stages of liver disease, even before reaching cirrhosis. Neuroinflammation occurs earlier in the molecular layer than in white matter, and is associated with infiltration of peripheral Th17 and Tfh lymphocytes.

## Introduction

Patients with liver disease may suffer hepatic encephalopathy (HE) with a wide range of neurological and psychiatric alterations ranging from mild cognitive impairment to coma and death^1–4^. Hyperammonemia and inflammation play synergistic roles in inducing the neurological alterations in HE^5–10^. The joint presence of inflammation and hyperammonemia is enough to induce mild cognitive impairment, even in the absence of liver failure^7^. These studies support that in chronic liver diseases peripheral inflammation contributes to cognitive and motor alterations in HE.

Studies in animal models show that cognitive and motor alterations in HE are a consequence of neuroinflammation, with microglia and astrocyte activation, which alters neurotransmission, leading to cognitive and motor impairment^11–17^. Moreover, blocking peripheral inflammation with anti-TNFa prevents microglia and astrocyte activation and cognitive and motor alterations in rats with HE^18,19^. This supports the idea that peripheral inflammation induces HE by activating microglia and astrocytes. This is also supported by studies showing that specific alterations of the immune system are associated with appearance of the neurological alterations in patients with minimal HE^20^. This may be mediated by different mechanisms. A main mechanism is by infiltration of peripheral lymphocytes into the brain^21^.

A few reports support the presence of neuroinflammation in patients with chronic liver diseases. Cagnin *et al*.^22^ showed by positron emission tomography that cirrhotic patients with HE show increased binding of [^11^C]-PK11195 to TSPO in brain, suggesting the presence of neuroinflammation^22^.

Dennis *et al*.^23^ performed immunohistochemistry studies in postmortem brain tissue from alcoholics with cirrhosis with and without HE and found activated microglia in about half of the patients with HE. Zemtsova *et al*.^24^ also analyzed neuroinflammation in post mortem brain tissue from patients with cirrhosis with and without HE and found an up-regulation of the microglial activation marker Iba-1 in the cerebral cortex from patients with HE^24^.

However, there are several questions related with neroinflammation in chronic liver disease which remain unclear. Studies in animal models of hyperammonemia and HE show that neuroinflammation is stronger in cerebellum than in other brain areas^11^. However, the above studies in cirrhotic patients have focused mainly in cerebral cortex, which is less affected in the rat models^11^. It is very likely that neuroinflammation would be also stronger in cerebellum than in other areas in patients with liver disease.

Moreover, patients with non-alcoholic steatohepatitis (NASH), without liver cirrhosis but with high levels of hyperammonemia and inflammation show cognitive impairment^7^. This suggests that neuroinflammation could be already present in patients with steatohepatitis. However, no studies on neuroinflammation have been performed in patients with steatohepatitis.

Also the possible relationship between cerebral infiltration of peripheral lymphocytes and glial activation in chronic liver disease remains unexplored, as well as the relationship between glial activation and neurodegeneration in these patients.

The aim of this work was to perform a detailed analysis by immunohistochemistry of the cerebellum of patients who, at the time of death, suffered from different grades of liver disease, from mild steatohepatitis to cirrhosis and HE of: (a) neuronal density in the Purkinje and granular layers; (b) microglial activation; (c) astrocyte activation; (d) infiltration of peripheral lymphocytes; (e) identification of the subtypes of lymphocytes infiltrated.

## Results

### Patients with SH1 stage of steatohepatitis already show neuronal loss in cerebellum

**Figure 1:**
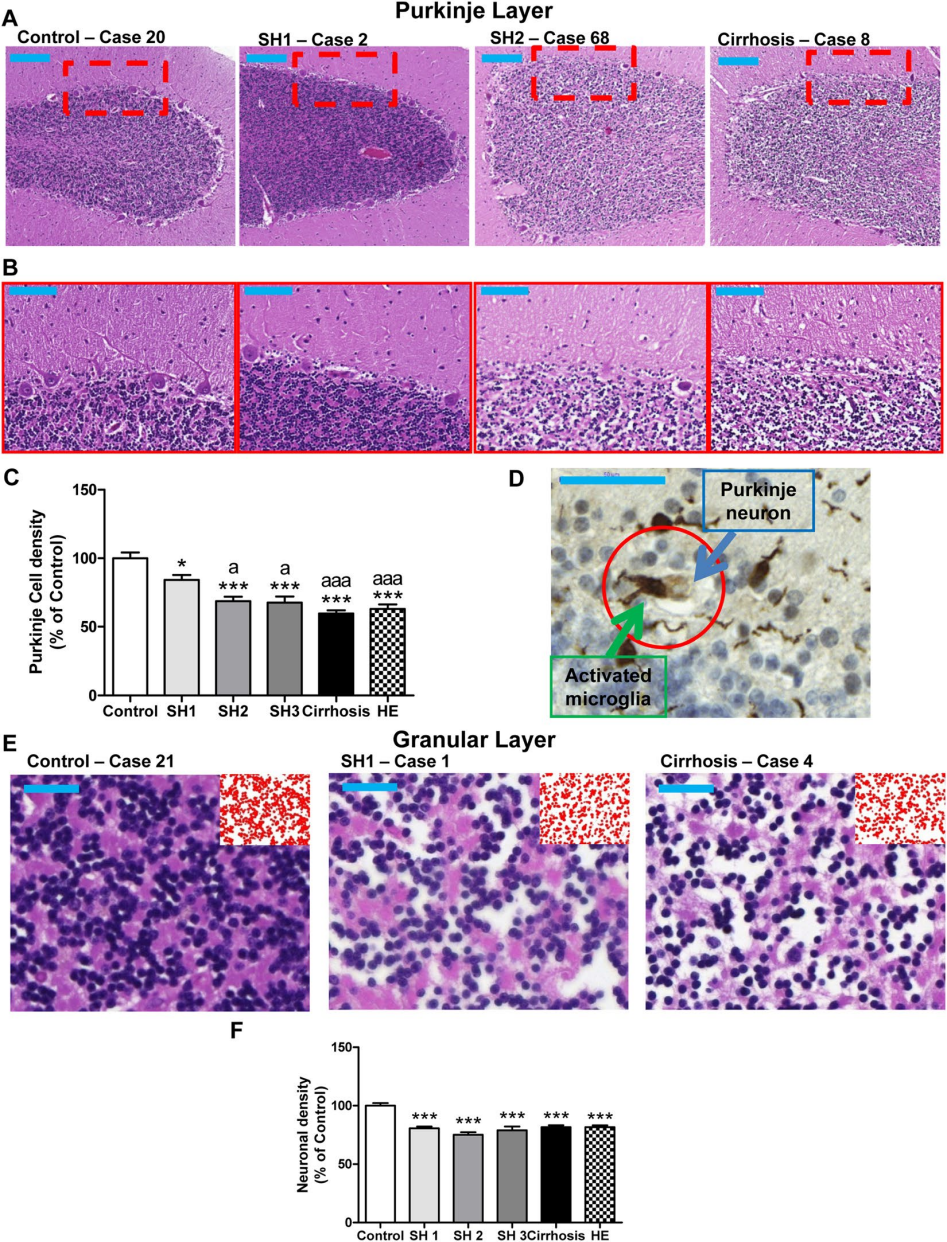
Patients with early stage (SH1) steatohepatitis already show neuronal loss in the Purkinje and granular layers. H&E stain was performed as described in methods. (**A**) Low magnification (5×; bar 200 μm) and (**B**) high magnification (20×; bar 100 μm) representative images of Purkinje layer and 56× images of granular layer (**E**). Neuronal density was quantified (**C** and **F**). (**D**) Shows a possible case of phagoptosis, where a Purkinje cell is phagocytosed by an activated microglial cell (bar 50 μm). One-way ANOVA with Bonferroni post-hoc test was performed to compare all groups. Values are the mean ± SEM of 4–9 individuals per group. Values significantly different from controls are indicated by asterisks and from SH1 patients by a. ***p < 0.005; ^a^p < 0.05; ^aaa^p < 0.005.

**Figure 2:**
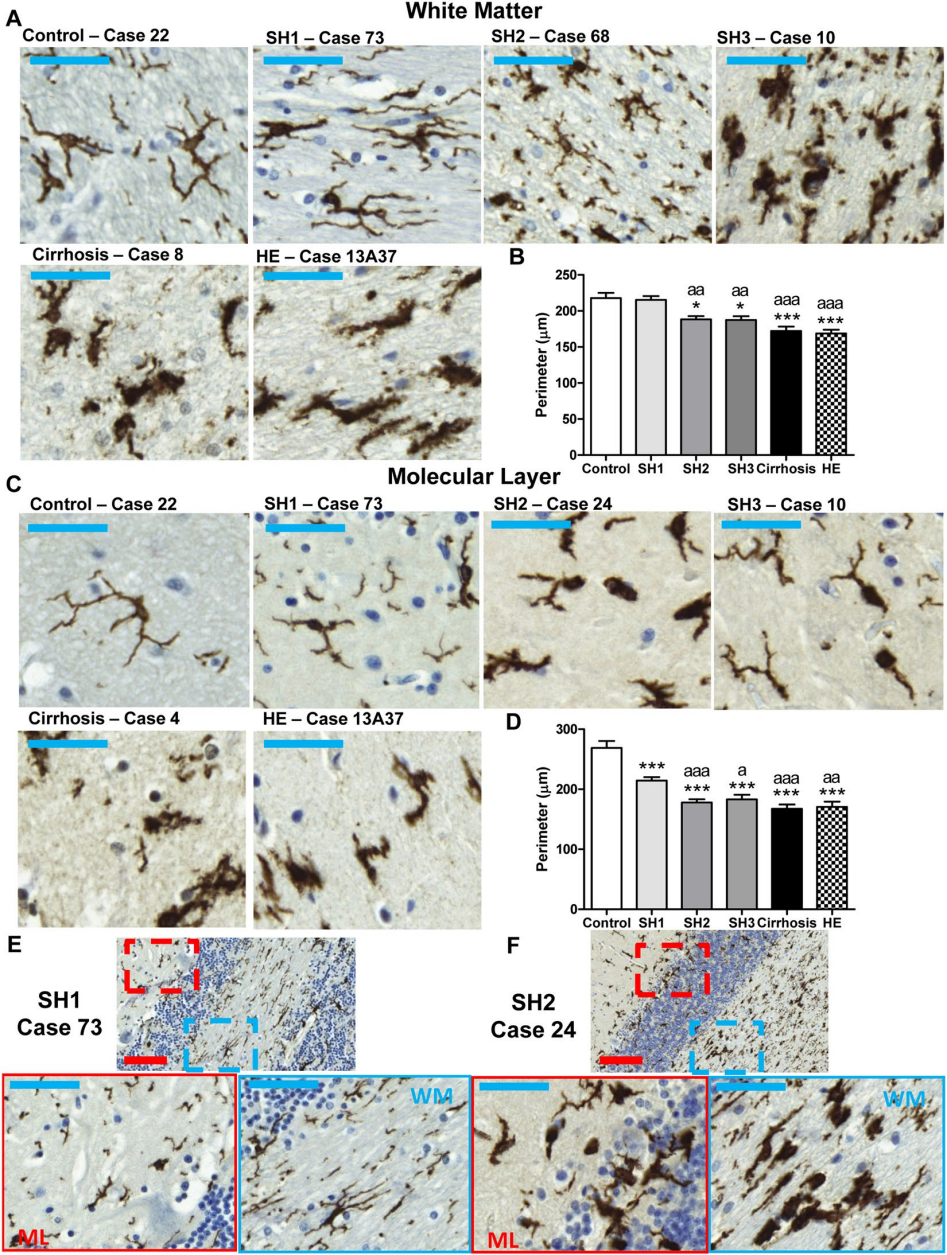
Patients with SH1 show microglial activation in molecular layer but not in white matter. Patients with more severe liver disease show microglial activation in both layers. Immunohistochemistry was performed using an antibody against Iba-1. Representative images of white matter (**A**) and molecular layer (**C**) are shown for patients with different grades of liver disease. Images showing both the molecular layer (ML, in red) and white matter (WM, in blue) at stages SH1 (**E**) and SH2 (**F**) of steatohepatitis are also shown. The perimeter of microglial cells was quantified in the molecular layer (**B**) and white matter (**D**). One-way ANOVA with Bonferroni post-hoc test was performed to compare all groups. Values are the mean ± SEM of 4–9 individuals per group. Values significantly different from controls are indicated by asterisks and from SH1 patients by a. *p < 0.05; ***p < 0.005; ^a^p < 0.05; ^aa^p < 0.001; ^aaa^p < 0.005. bar in blue = 50 μm; bar in red = 100 μm.

Hematoxylin-Eosin stain shows neuronal loss in Purkinje (Fig. 1A,B) and granular layers (Fig. 1E) of patients with different grades of liver disease. The number of Purkinje neurons is reduced (84 ± 4%; p < 0.05) in patients with SH1 compared to controls (100 ± 4%). The neuronal loss progresses with the steatohepatitis stages: SH2 (69 ± 3%, p < 0.005) and SH3 (68 ± 5% of controls; p < 0.005). Patients with liver cirrhosis and with HE show even more neurodegeneration, reducing the number of Purkinje neurons to 60 ± 2% (p < 0.005) and 63 ± 3% (p < 0.005) of controls (Fig. 2C). Figure 1D shows an image where a microglial cell may be “eating” a Purkinje neuron. This suggests that activated microglia in the molecular layer could contribute to Purkinje neurons loss.

Neuronal loss was also observed in the granular layer (Fig. 1E). SH1 patients already show reduced neuronal density (81 ± 2%; p < 0.005) compared to controls (100 ± 2%), which remain similar in SH2 (75 ± 2%), SH3 (79 ± 3%) and in patients with liver cirrhosis (82 ± 2%) and HE (82 ± 2%) (Fig. 1F).

### Microglial activation occurs earlier in molecular layer than in white matter in patients with steatohepatitis

Figure 2 shows microglia in white matter and molecular layer. At stage SH1 of steatohepatitis, the morphology of microglia remains unaltered compared to controls in white matter (Fig. 2A, SH1 and Fig. 2B) while it is already activated in the molecular layer (Fig. 2C, SH1 and Fig. 2D).

In white matter, the perimeter of microglia (Fig. 2B), a measure of the grade of activation, was reduced (p < 0.05) in SH2 and SH3 patients (188 ± 5 and 187 ± 5 µm, respectively) compared to controls (218 ± 7 µm) and SH1 patients (215 ± 5 µm). The activation increased in liver cirrhosis (172 ± 6 µm; p < 0.005) and HE (169 ± 5 µm; p < 0.005).

In the molecular layer, microglial activation occurs earlier than in white matter. SH1 patients already show activated microglia with a reduced perimeter (214 ± 6 µm; p < 0.005) compared to controls (259 ± 12 µm). Microglial activation increased with progression of liver disease as reflected by the reduction of microglial perimeter (Fig. 2C and D): patients with SH2 (178 ± 6 µm); SH3 (183 ± 8 µm); liver cirrhosis (167 ± 7 µm) and HE (170 ± 9 µm).

Figure 2E shows an image of both the molecular layer (ML, in red) and white matter (WM, in blue) at stage SH1 of steatohepatitis. The morphology of microglia remains ramified and unaltered compared to controls in white matter while it is already activated in the molecular layer, with reduced ramifications and ameboid shape. Figure 2F shows a similar image in a patient at stage SH2. In this case microglia is activated both in molecular layer and white matter. This suggests that activation of microglia in liver disease begins in the molecular layer and appears later in the white matter.

**Figure 3:**
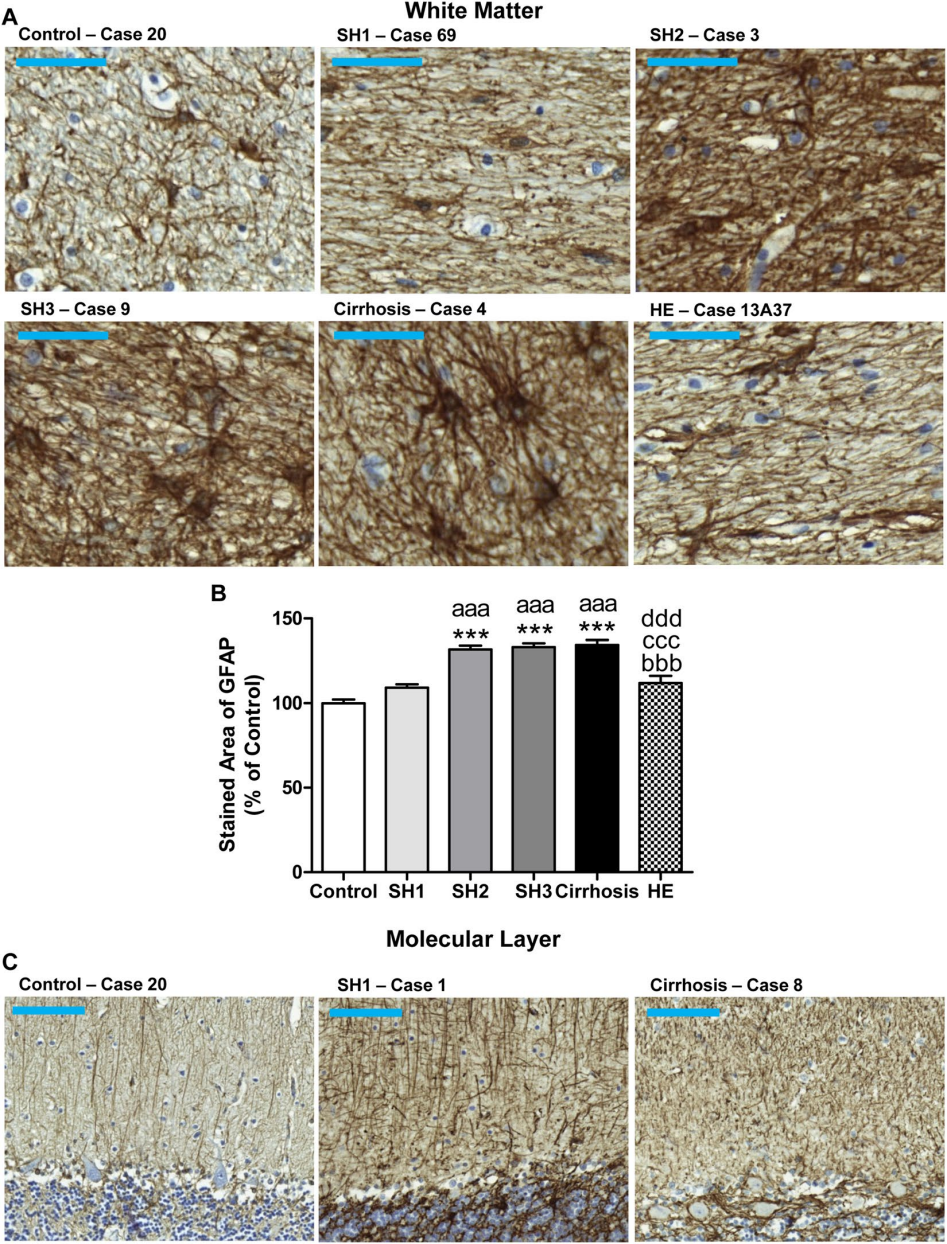
Patients with SH2 steatohepatitis or more severe liver failure show astrocytes activation in white matter and degeneration of fibers of Bergmann glia. Immunohistochemistry was performed using an antibody against GFAP. Representative images of white matter astrocytes (**A**; bar 50 μm) and Bergmann glial fibers morphology in molecular layer (**C**; bar 100 μm) are shown for patients with different grades of liver disease. The area stained by GFAP was quantified and is expressed as percentage of controls (**B**). One-way ANOVA with Bonferroni post-hoc test was performed to compare all groups. Values are the mean ± SEM of 4–9 individuals per group. Values significantly different from controls are indicated by asterisks, from SH1 patients by a, from SH2 patients by b, from SH3 patients by c and from cirrhotic patients by d. ***p < 0.005; ^aaa^p < 0.005; ^bbb^p < 0.005; ^ccc^p < 0.005; ^ddd^p < 0.005.

As occurs for microglial activation, in white matter astrocyte activation is not present at stage SH1 and starts in patients with SH2 (Fig. 3A and B), who show increased area stained by GFAP (132 ± 2%; p < 0.005) compared to controls (100 ± 2%). A similar astrocyte activation was observed in SH3 (133 ± 2%; p < 0.005) and cirrhotic patients (134 ± 3%; p < 0.005). Astrocyte activation was lower in patients with HE (112 ± 4%).

We also analyzed Bergmann glia, a subtype of cerebellar astrocytes that reside in molecular layer next to Purkinje neurons. Bergmann glial fibers stained with GFAP present a disorganized, damaged and hypertrophied morphology in all groups of patients with steatohepatitis, cirrhosis and HE compared to controls, who show thin and intact morphology (Fig. 3C).

### Patients with steatohepatitis show infiltration of T but not B lymphocytes

**Figure 4:**
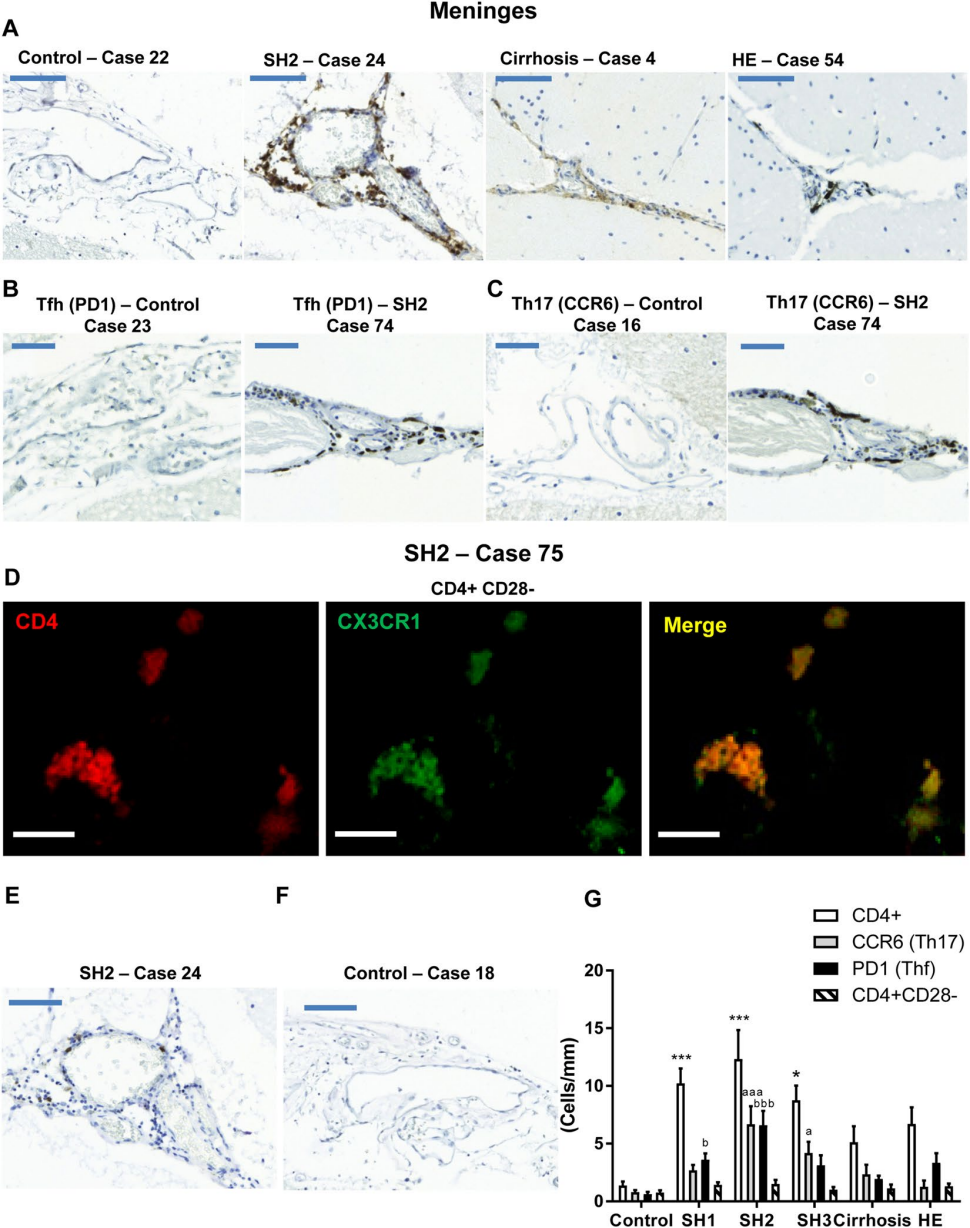
Patients with steatohepatitis show T lymphocytes infiltration in meninges. Representative images of sections stained using an antibody against an specific marker for T lymphocytes (CD4) are shown in (**A**) for patients with different grades of liver disease (bar 100 μm). Representative images of sections stained with Tfh and Th17 subtype markers are shown in (**B** and **C**), respectively for a control subject and a patient with SH2 (bar 50 μm). D shows a double immunofluorescence (CD4 in red and CX3CR1 in green) showing a SH2 patient with infiltration of CD4^+^CD28^−^ T lymphocytes in meninges (bar 25 μm). E shows a representative image stained with anti-CD20, a marker of B lymphocytes, in a section of the same SH2 patient shown in A for T lymphocytes. F shows a representative image of a control patient stained with anti-CD20. The number of CD4^+^, Tfh, Th17 and CD4^+^CD28^−^ T lymphocytes was quantified in patients with different grades of liver disease (**G**). One-way ANOVA with Bonferroni post-hoc test was performed to compare all groups. Values are the mean ± SEM of 4–9 individuals per group. Values significantly different from controls are indicated by asterisks, from SH1 patients by a and from SH2 patients by b. *p < 0.05; ***p < 0.005; ^a^p < 0.05; ^aaa^p < 0.005; ^b^p < 0.05; ^bbb^p < 0.005.

The analysis of peripheral immune cells infiltration shows an important presence of CD4^+^ cells (Fig. 4A) but not (or very few) CD20^+^ cells (Fig. 4E and F) in the meningeal space of cerebellum of patients with different stages of steatohepatitis.

SH1 patients already show CD4^+^ T lymphocytes infiltration (10 ± 1 cells/mm; p < 0.005) while controls show only 1 ± 1 cells/mm of meninges length. The infiltration of CD4^+^ T lymphocytes increased in SH2 patients to 12 ± 3 cells/mm (p < 0.005) and decreased thereafter with the grade of liver disease, reaching 9 ± 1 cells/mm in SH3 patients (p < 0.05); 5 ± 1 cells/mm in cirrhotic patients and 6 ± 1 cells/mm in patients with HE (Fig. 4G).

To assess which subtype of CD4^+^ T lymphocytes are infiltrating the cerebellar meninges we analyzed three subtypes: CD4^+^CD28^−^ (Fig. 4D), Th follicular (Tfh) (Fig. 4B) and Th17 lymphocytes (Fig. 4C). Most infiltrating lymphocytes are Tfh or Th17 (Fig. 4G). In SH1 patients, 3 ± 1 infiltrated cells/mm are Th17 and 4 ± 1 cells/mm are Tfh. In SH2 7 ± 2 infiltrated cells/mm are Th17 and 7 ± 1 cells/mm are Tfh. In SH3, Th17 cells represent nearly 50% of total infiltrated cells (4 ± 1 cells/mm), while Tfh cells are 3 ± 1/mm (Fig. 4G).

## Discussion

**Figure 5:**
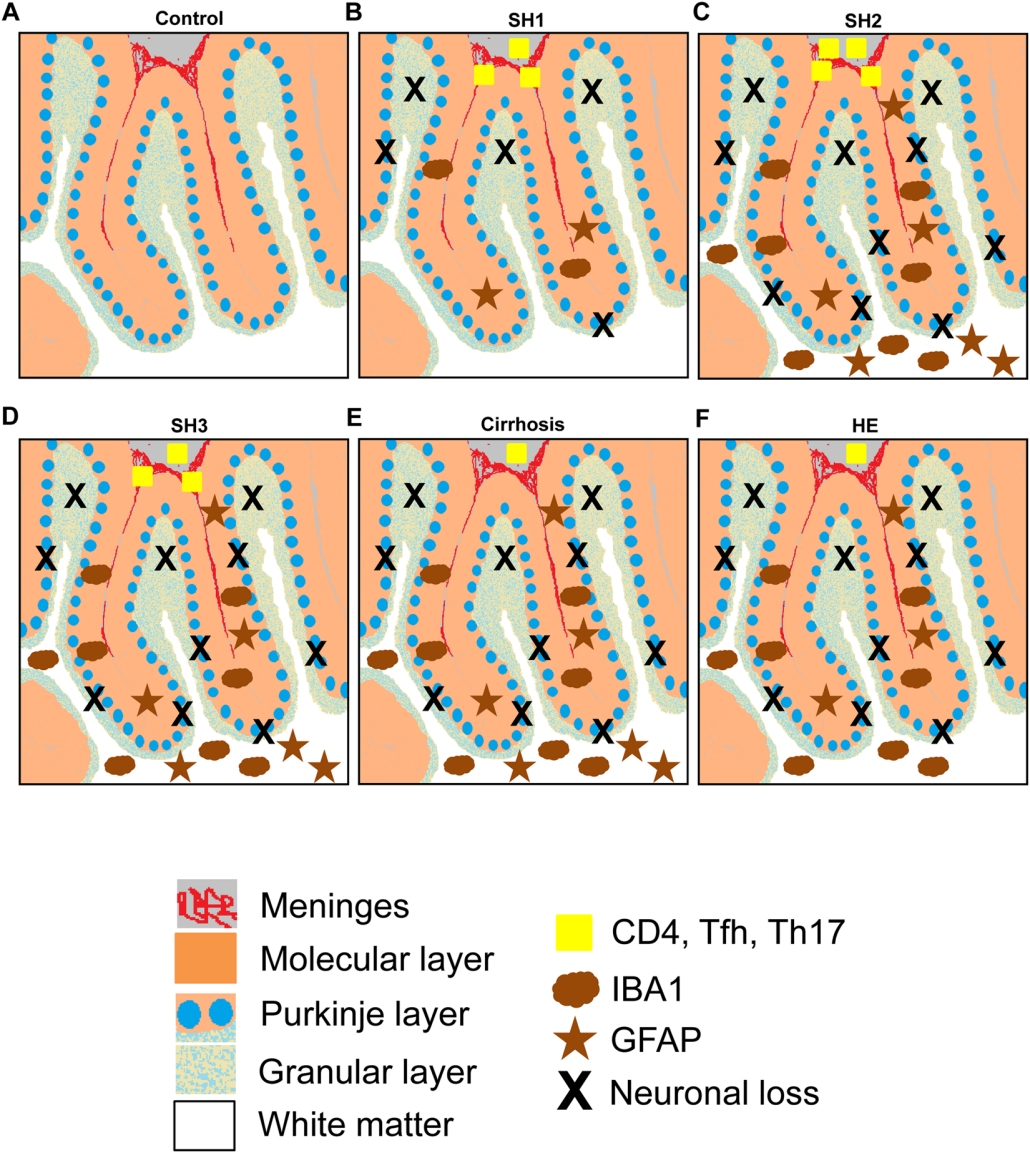
Scheme summarizing the histo-architectural changes in cerebellum of patients with different grades of liver disease. (**A**) Cerebellum of a control subject with normal liver. (**B**) Patients at early stage (SH1) of steatohepatitis. An infiltration of CD4^+^ T lymphocytes (yellow squares) is observed in meninges (in red). CD4^+^ cells activate microglia (brown clouds) in the molecular layer (orange layer) and alter Bergmann glial fiber morphology (brown stars). Microglia and astrocytes are not activated in white matter (white color). Activated microglia in the molecular layer induces neurodegeneration (black X) of Purkinje (blue circles) and granular neurons (light blue spotted layer). (**C**) Patients at SH2 stage. The changes observed in SH1 are exacerbated, with increased microglia and astrocyte activation in molecular layer (orange). Microglia (brown clouds) and astrocytes (brown stars) are also activated in white matter (in white) and neuronal loss (black X) increases in Purkinje layer (blue circles). (**D**) Patients at SH3 stage. The changes observed are similar to SH2 patients. The only difference is a small decrease in CD4^+^ T lymphocytes (yellow squares). (**E** and **F**) Patients with liver cirrhosis and HE. A decrease of CD4^+^ lymphocytes (yellow square) infiltration is observed. Chronic neuroinflammation increases neuronal loss (black X) in Purkinje layer (blue circles). A significant reduction of GFAP content in white matter of patients with HE is observed (brown stars).

**Figure 6:**
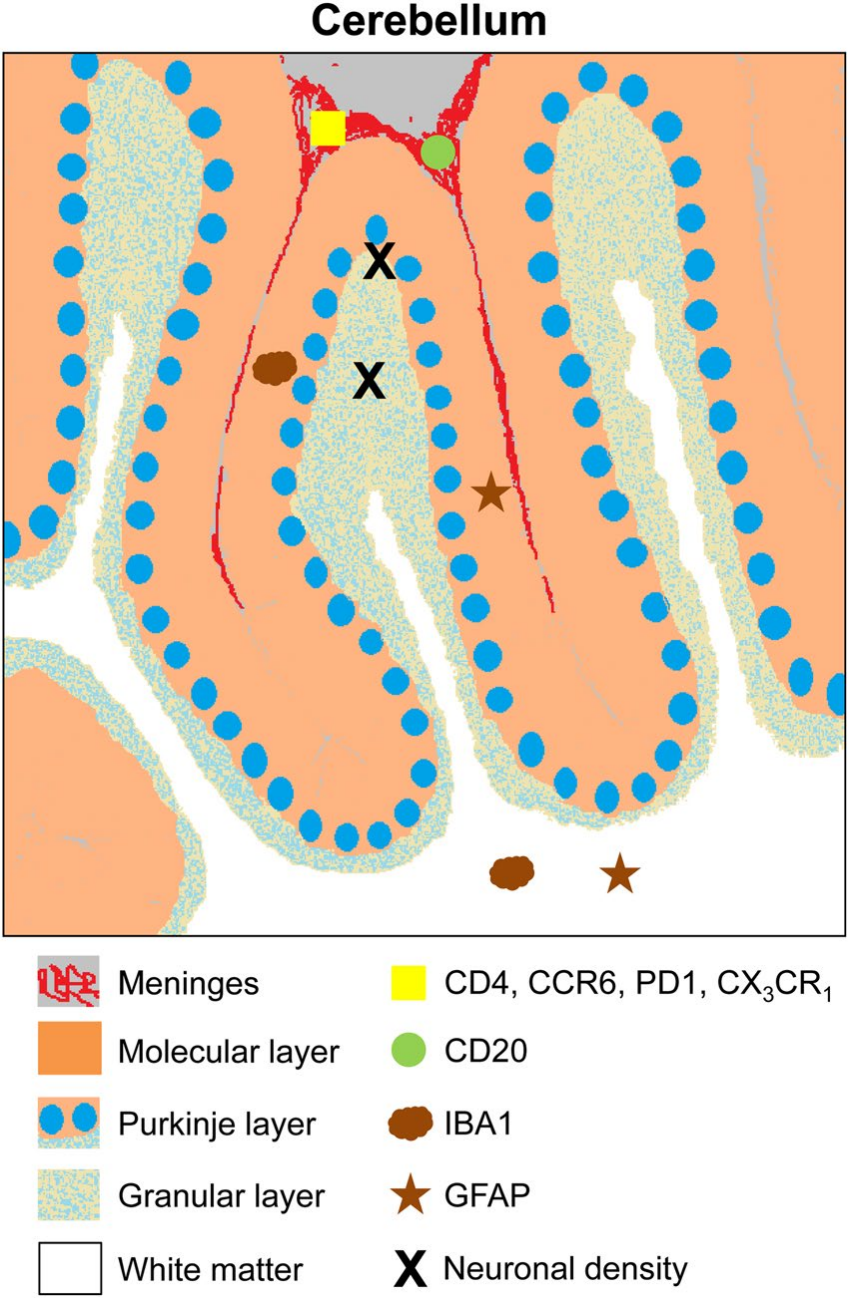
Scheme summarizing the analysis performed and their localization in cerebellum. Histo-architectural analysis was performed on post-mortem human cerebellum sections. Neuronal density analysis (black X) was performed in Purkinje layer (blue circles) and in granular layer (light blue spotted layer). Microglial (brown cloud, Iba1) and astroglial (brown star, GFAP) activation analysis was performed in molecular layer (in orange) and white matter (white). Infiltration of B lymphocytes (green circle, CD20) and of total T lymphocytes (yellow square, CD4) and the T lymphocytes subtypes Tfh, Th17 and CD4^+^CD28^−^ was analyzed in meninges (in red).

The results reported show that patients with different grades of liver disease show T lymphocytes infiltration in the meninges of cerebellum, associated with activation of microglia and astrocytes and loss of Purkinje and granular neurons. Another main finding of this study is that these alterations are already present in patients with steatohepatitis. The data reported suggest the sequence of events during progression of liver disease summarized in Fig. 5. At stage SH1 of steatohepatitis (Fig. 5B) there is an infiltration of T lymphocytes, mainly of the Tfh and Th17 subtypes, with the presence of some CD4^+^-CD28^−^ T lymphocytes. This is associated with microglia activation in the molecular layer while white matter remains unaffected. In the molecular layer Bergmann glia also seems to be damaged. In this SH1 stage there is already a small reduction in the number of Purkinje and granular neurons (Fig. 5B). At stages SH2 (Fig. 5C) and SH3 (Fig. 6D) of steatohepatitis the infiltration of T lymphocytes continues rising. Activation of microglia and astrocytes is extended to white matter and there is a further reduction in the number of Purkinje neurons (Fig. 5C and D). In patients with cirrhosis (Fig. 5E) and HE (Fig. 5F) there is a reduction in the number of infiltrating lymphocytes. Neuronal loss remains at levels similar to patients with SH2-SH3 (Fig. 5E and F) and microglia and astrocytes remain activated both in the molecular layer and white matter in patients with cirrhosis. Patients with HE show a decrease of content of GFAP in white matter (Fig. 5F). A down-regulation of GFAP has been already shown in patients with HE^25^ and in rat models of HE^26,27^. In cultured astrocytes, addition of ammonia or of TNFa also induces down-regulation of both mRNA for GFAP and GFAP protein level^28,29^. Increased ammonia and/or TNFa levels in cirrhotic patients with HE could contribute to the remarkable reduction in GFAP content.

These data show that neuroinflammation in cerebellum occurs at early stages of liver disease, even before reaching cirrhosis.

Previous studies on microglial activation have been performed mainly in patients with stablished liver cirrhosis and have focused mainly in cerebral cortex and periventricular region^23,24^. Dennis *et al*.^23^ reported microglial activation in white matter and gray matter and dystrophic microglia in white matter in the periventricular region in patients with liver cirrhosis. They also showed increased microglial proliferation in a subset of patients with HE and a small decrease in neuronal density in the superior frontal gyrus of patients with HE^23^. Zemtsova *et al*.^24^ reported that microglia is activated in cirrhotic patients with HE but not in those without HE^24^. However, it must be taken into account that microglial activation was quantified by western blot analysis of Iba1 content in cerebral cortex. This procedure is not sensitive enough to detect microglial activation. Moreover, the analysis was performed only in cerebral cortex, which is much less sensitive than cerebellum to neuroinflammation in hyperammonemia and chronic liver failure^11^. Rats with chronic hyperammonemia or with liver failure and HE show strong neuroinflammation and microglial activation in cerebellum but not in prefrontal cortex, which remains unaltered^11^. The focus of previous studies in cerebral cortex may explain the milder microglial activation reported compared with the present work in cerebellum, which is much more strongly affected.

The induction of neuroinflammation in cerebellum at early stages of liver disease is in agreement with reports showing that other cerebellar alterations also occur at early stages of liver disease. For example, non-invasive blood flow measurement in cerebellum detects minimal HE (MHE) earlier than psychometric tests^30^. Blood flow was increased in cerebellar hemisphere (but not in other brain regions) in cirrhotic patients with or without MHE, indicating that cerebellum is the more sensitive area, and that functional alterations in cerebellum occur before they are reflected in impairment of the neurological functions evaluated by the PHES battery of psychometric tests used to detect MHE^30^. The earliest alteration of cerebellum is supported by reports showing that functions modulated by cerebellum are impaired in patients classified as without MHE by the PHES battery. Butz *et al*.^31^ showed that ataxia, tremor, and slowing of finger movements are early markers for cerebral dysfunction in HE patients before alterations becoming detectable with PHES. Bimanual and visuo-motor coordination are also impaired in patients without MHE according to the PHES^32,33^. All these motor functions are modulated in cerebellum, supporting that cerebellum is the earliest brain area affected in liver disease. This agrees with the strong neuroinflammation observed here in cerebellum at early stages of liver disease.

Another main finding of this work is the fact that lymphocytes infiltration, glial activation and neuronal loss may be observed already in patients at early stages of steatohepatitis. Although HE and MHE are usually evaluated in patients with liver cirrhosis, MHE has also been reported in patients with NASH^7^. This agrees with the increased neuroinflammation found here in cerebellum of patients with steatohepatitis, even before reaching cirrhosis.

The data reported also show neuronal loss in the Purkinje and granular layers of cerebellum already in patients at early stages of steatohepatitis. It is generally considered that liver failure is not associated with neurodegeneration or neuronal loss. However, a good number of reports show neuronal loss, especially in cerebellum. This has been reported in the so called “acquired hepatocerebral degeneration”, with neuronal loss in basal ganglia, cerebral cortex and cerebellum^34^. Cerebellar degeneration, with loss of Purkinje neurons was also reported in 20 out of 36 patients who died with liver cirrhosis and HE^35^. Neuronal loss, especially in cerebellum, is therefore more frequent than assumed in liver disease. We show here for the first time that Purkinje and granular neurons loss also occurs in cerebellum already at early stages of steatohepatitis.

The neuronal loss in liver disease may explain why the recovery of neurological functions is not complete after liver transplantation in patients^36,37^. Mechtcheriakov *et al*.^36^ reported that improvement of visuo-motor deficits after liver transplantation is incomplete in patients with MHE. Visuo-motor coordination is modulated in cerebellum. The loss of Purkinje and granular neurons observed here in liver disease will not recover after liver transplantation and may explain the incomplete recovery of some functions modulated in cerebellum.

The present report also provides some new insights on the mechanisms underlying neuronal loss and neuroinflammation in chronic liver disease. We show in Fig. 1D a microglia cell which seems to be phagocytosing a Purkinje neuron. Microglia removes dead and dying neurons. However, microglia can also phagocytose live neurons, contributing to neuronal cell loss. This process has been named as “phagoptosis”^38^ and may be triggered by inflammation^38^. We have observed a relevant number of images of Purkinje neurons which seem to be phagocytosed by microglia. This suggests that “phagoptosis” could contribute to neuronal loss in cerebellum in chronic liver disease.

The results reported also suggest that infiltration of T lymphocytes would contribute to glial activation in the molecular layer already at SH1. We do not observe relevant infiltration of B lymphocytes.

Infiltration of lymphocytes in the meningeal space also occurs in patients with multiple sclerosis (MS), contributing to neuroinflammatory processes at early stages of MS and correlating with accelerated clinical disease progression^39^. As shown here for patients with chronic liver disease, Th17^40^ and Tfh^41^ lymphocytes also constitute a relevant part of infiltrated lymphocytes in MS, playing a role in its pathogenesis^39–41^. However, in MS, meningeal infiltrates also contain large amounts of B lymphocytes and form follicle-like structures which contribute to demyelination^39^, while we did not find relevant amounts of B lymphocytes in patients with chronic liver disease. This may explain the different structural and neurological consequences in MS and liver disease.

We observe infiltration mainly of Th17 and Tfh lymphocytes. To infiltrate the brain, T lymphocytes must be first activated in peripheral blood. Once activated, they divide and differentiate into one of several subtypes, including Th1, Th2, Th17, Th22, iTreg or Tfh, which secrete different cytokines to facilitate different types of immune responses^42^. The infiltration of Th17 and Tfh lymphocytes in meninges suggest that, in chronic liver disease, activated T lymphocytes differentiate mainly to Th17 and Tfh. In agreement with this possibility, an expansion of Th17 and Tfh lymphocytes has already been reported in primary biliary cirrhosis^43^. Th17 are also expanded in patients with non-alcoholic fatty liver disease, infiltrate the liver and correlate with progression to steatohepatitis^44^. We show here that Th17 infiltrate also the meninges in patients with steatohepatitis. In experimental autoimmune encephalitis, a model of multiple sclerosis, Th17 cells induce changes in the meningeal microenvironment (cytokines, chemokines, proteins) that support the recruitment and maintenance of inflammatory cells in brain^45^. Infiltration of Th17 in the meninges would be therefore a main contributor to induce and maintain the neuroinflammation in cerebellum of patients with steatohepatitis and liver cirrhosis.

In summary, we show that patients with chronic liver disease show infiltration of Th17 and Tfh lymphocytes in the meninges in cerebellum already at early stages of steatohepatitis. T lymphocytes infiltration is associated with microglia and astroglia activation, first in the molecular layer and subsequently in white matter of cerebellum. Glial activation is associated with loss of Purkinje and granular neurons. To this neuronal loss may contribute both activated microglia, by phagocytosing neurons, and activated astrocytes, which we found are damaged, reducing their ability to support neuronal cell survival.

The results reported here, together with the previous paper showing that 5 out of 11 patients with NASH show mild cognitive impairment^7^ suggest that it would be worth to perform psychometric tests in patients with steatohepatitis to assess if they have MHE. If this is the case, patients should be treated to reverse MHE and prevent worsening of neurological condition. In addition to the PHES battery it would be convenient to perform tests assessing functions modulated in cerebellum such as visuo-motor or bimanual coordination. Early detection of MHE would allow treating the patients and improving, preventing or delaying neurological impairment.

## Methods

### Cerebellum and liver tissue

Post-mortem human brain and liver samples were obtained from Instituto Medicina Legal y Ciencias Forenses (Valencia) and 3 Biobanks in Spain: Hospital Universitario Fundación Alcorcon; A Coruña and Hospital Clinic IDIBAPS, Barcelona. For each case appropriate authorization to collect the tissue for research was obtained. Subjects were classified in four groups: control, steatohepatitis, cirrhosis and HE. Steatohepatitis group was subdivided in three subgroups (SH1, SH2 and SH3) depending on severity (see below). Samples were from 34 individuals: 6 controls, 9 SH1, 6 SH2, 4 SH3, 4 cirrhotics and 5 cirrhotic with HE. The characteristics of the subjects, cause of death and post-mortem time to collect samples are detailed in Table 1. Sections of cerebellum and liver were fixed in 10% formalin for 96 h at 4 °C and processed for paraffin embedding.

**Table 1:** Characteristics and source of the samples

CASE	GRADE	SEX	AGE	CAUSE OF DEATH	PMD (h)	BIOBANC
**16**	Control	M	79	N.A.	2	FHU
**18**	Control	M	76	N.A.	5	FHU
**20**	Control	M	62	Mesenteric ischemia	14	FHU
**21**	Control	M	72	N.A	18	FHU
**22**	Control	M	60	Bronchoaspiration	N.A.	FHU
**23**	Control	M	67	N.A	N.A.	FHU
**1**	SH1	M	46	Ischemic cardiopathy	11	IML
**2**	SH1	M	49	Ischemic cardiopathy	14	IML
**7**	SH1	M	36	Asphyxia from hanging	14	IML
**69**	SH1	M	46	Sudden cardiac death	48	IML
**70**	SH1	M	50	Sudden cardiac death	36	IML
**71**	SH1	M	53	Sudden cardiac death	36	IML
**72**	SH1	F	47	Sudden cardiac death	24	IML
**73**	SH1	M	45	Sudden cardiac death	24	IML
**78**	SH1	M	51	Sudden cardiac death	40	IML
**3**	SH2	F	70	Lower gastrointestinal bleeding	12–24	IML
**24**	SH2	M	40	N.A	12–24	IML
**68**	SH2	M	55	Sudden cardiac death	18	IML
**74**	SH2	M	48	Sudden cardiac death	24	IML
**75**	SH2	M	30	Sudden cardiac death	18	IML
**76**	SH2	M	45	Sudden cardiac death	18	IML
**5**	SH3	M	49	Severe ischemic cardiopathy	12–24	IML
**9**	SH3	M	44	Alcohol-drug adverse reaction	36	IML
**10**	SH3	M	49	N.A	26	IML
**77**	SH3	M	47	Sudden cardiac death	20	IML
**4**	Cirrhosis	M	53	Gastrointestinal bleeding	12–24	IML
**8**	Cirrhosis	M	69	Gastrointestinal bleeding	17	IML
**15A51**	Cirrhosis	N.A.	N.A.	Intra-abdominal sepsis	N.A.	A Coruña
**58**	Cirrhosis	M	60	Sudden cardiac death	5	IDIBAPS
**54**	Cirrhosis + HE	F	78	Cardiorespiratory arrest	9,5	IDIBAPS
**55**	Cirrhosis + HE	M	76	Acute parotitis	3,5	IDIBAPS
**56**	Cirrhosis + HE	M	58	Acute respiratory failure	5	IDIBAPS
**57**	Cirrhosis + HE	F	70	Sudden cardiac death	3,5	IDIBAPS
**13A37**	Cirrhosis + HE	M	54	Metabolic coma	N.A.	A Coruña

IML: Instituto Medicina Legal y Ciencias Forenses Valencia; FHU: Biobanco Hospital Universitario Fundación Alcorcon; A Coruña: Biobanco A Coruña; IDIBAPS: Biobanc Hospital Clinic IDIBAPS, Barcelona.

N.A.: Data not available.

### Liver histology

The grade of liver disease was established using hematoxilin-eosin and Masson trichrome stains of formalin-fixed paraffin-embedded liver. Steatohepatitis was graded by experienced pathologists using a scoring system as in^46^. The histological features were grouped into four categories: steatosis, inflammation, fibrosis and hepatocellular injury. The score was defined as the sum of scores for steatosis grade(0–3), steatosis location(0–3), lobular inflammation(0–3), fibrosis stage(0–3) and presence of necrosis(0–1). Subjects that score <3 or less than 5% steatosis were classified as controls, between 3–6 as SH1, between 7–9 as SH2 and patients that score >9 were classified as SH3.

### Cerebellum histology and immunohistochemistry

Five-micrometer thick, paraffin-embedded sections were cut and mounted on coated slide glass. Sections were rehydrated and antigen retrieved with the Dako 3 in 1 AR buffer EDTA pH 9.0 in a DAKO PT link. The tissue sections were then processed with the Envision Flex + kit (DAKO) blocking endogenous peroxidase activity for 5 min and then incubating with primary antibody. The reaction was visualized by incubating with Envision Flex + horseradish peroxidase for 20 min and finally with diaminobenzidine for 10 min. Sections were counterstained with Mayer’s hematoxylin (DAKO S3309; Ready to use) for 5 min. The analyses performed and their localization in cerebellum are summarized in Fig. 6.

The primary antibodies used were: anti-Iba1 as microglial marker (Wako, 019-19741; 1:300 for 30 min), anti-GFAP as astrocyte marker (DAKO, IR524; ready to use for 20 min), anti CD4 for T lymphocyte staining (DAKO, M7310, 1:50 for 20 min), anti CD20 for B lymphocyte detection (DAKO, IR604, ready to use for 20 min), anti PD1 (Abcam, ab52587, 1:100 for 30 min) as Tfh cell marker and anti CCR6 as Th17 cell marker (R&D System, MAB195, 1:150 for 30 min).

For histological analysis of cerebellum, Hematoxylin and Eosin (H&E) stain was performed.

### Double Immunofluorescence analysis of CD4 and CX_3_CR_1_

For detection of CD4^+^CD28^−^ lymphocytes a double immunofluorescence was performed, using anti-CD4 and anti CX_3_CR_1_ antibodies. Five-micrometer thick, paraffin-embedded sections were cut and mounted on coated slide glass. Sections were rehydrated and antigen retrieved with the Dako 3 in 1 AR buffer EDTA pH 9.0 in a DAKO PT link. Sections were processed with 0,1% Sudan Black B (SIGMA) in 70% EtOH for 20 min for masking fluorescent tissue components and then blocked with normal goat serum (because the secondary antibodies were from goat), before being incubated overnight with primary antibodies (CD4, 1:50 CX_3_CR_1_, 1:100) from Abcam (ab846 and ab8021, respectively), diluted in blocking buffer. Secondary fluorescent antibody (Goat anti-Rabbit 488, 1:500 and Goat anti-Mouse 555, 1:500) from Alexa were used. The nuclei were stained with DAPI (Sigma-Aldrich). The images were observed under confocal microscope (Leica TCS-SP2-AOBS) and photographically recorded.

Immunohistochemical quantification was performed using ImageJ (1.48 v). For analysis of microglial activation and stained areas by GFAP antibody a single section measuring 3 cm^2^ was analyzed from each case. The analysis of microglial activation and GFAP stained areas was performed in white matter and molecular layer of cerebellum. Using Auto Local Threshold and analyze particles functions the intensity thresholds and size filter were applied.

To measure the perimeter of microglia in white matter and molecular layer of cerebellum, the Bernsen method was used and 2000–20000 size filter was applied. For each case and region at least 10 fields (56×) were photographed and 30–40 cells were quantified. The 10 fields were randomly chosen to cover most of the areas of white matter and molecular layer. The results were then converted from pixels to micrometers.

For GFAP staining no size filter was applied. For each case and region at least 10 fields (56×) were randomly photographed and the total GFAP stained area was quantified. The results were expressed as percentage of total GFAP stained area.

Analysis of CD4, Tfh and Th17 cells was performed in meningeal space of cerebellum. For each case 10 fields (20×) were randomly photographed. Positive cells were manually counted and length of meninges measured using ImageJ. The results were expressed as cells/mm.

### Neuronal density analysis

For quantification of neuronal density in granular layer and Purkinje layer of cerebellum H&E stained sections were used. Granular cell’s density was quantified using Auto Local Threshold and analyze particles functions of ImageJ. To select granular cells, Intermode method was used and no size filter was applied. At least 10 random fields (56×) for each case were quantified. Results were expressed in percentage related to the mean of the control subjects, considered 100%.

For Purkinje neurons density analysis at least 10 random fields (7×) for each case were quantified. Purkinje neurons were manually counted using ImageJ and results were expressed in percentage related to the mean of the control cases, considered 100%.

### Statistical analysis

Results are expressed as mean ± SEM. Data were analyzed by one-way ANOVA followed by Bonferroni Post hoc test. P < 0.05 is considered significant differences.
